# Molecular epidemiology of levofloxacin-resistant *Klebsiella pneumoniae* and the association of plasmid-mediated quinolone resistance genes with key biological phenotypes

**DOI:** 10.1128/spectrum.01031-26

**Published:** 2026-05-20

**Authors:** Qian Zhang, Xinmiao Jia, Shiyu Chen, Peiyao Jia, Ying Zhu, Wei Yu, Xiaobing Chu, Zongping Li, Qiwen Yang

**Affiliations:** 1 Department of Clinical Laboratory, State Key Laboratory of Complex Severe and Rare Diseases, Peking Union Medical College Hospital, Chinese Academy of Medical Sciences and Peking Union Medical College 34732, Beijing, China; 2 Peking Union Medical College, Chinese Academy of Medical Sciences, Peking Union Medical College Hospital 34732, Beijing, China; 3 Center for bioinformatics, National Infrastructures for Translational Medicine, Institute of Clinical Medicine & Peking Union Medical College Hospital, Chinese Academy of Medical Sciences and Peking Union Medical College 34732, Beijing, China; 4 Key Laboratory of Pathogen Infection Prevention and Control (Peking Union Medical College), Ministry of Education 569810, Beijing, China; Emory University School of Medicine, Atlanta, George, USA

**Keywords:** *Klebsiella pneumoniae*, PMQR, QRDR, quinolone resistance, epidemiology

## Abstract

*Klebsiella pneumoniae* is a major opportunistic pathogen in China, yet the molecular epidemiology of quinolone resistance remains poorly characterized. This study analyzed 2,433 clinical isolates from 37 Chinese hospitals (2018–2022). The overall levofloxacin-non-susceptible (NS) rate was 53.60%, with urinary tract isolates showing higher resistance. Whole-genome sequencing identified 12 plasmid-mediated quinolone resistance (PMQR) genes. Among 1,304 NS strains, 74.54% carried at least one PMQR gene (mainly *qnrS*, *qnrB*, and *aac(6*′*)-Ib-cr*), and 60.20% also had quinolone resistance-determining region (QRDR) mutations. Functional studies revealed diverse phenotypic impacts. Most PMQR genes conferred low-level resistance (minimum inhibitory concentration [MIC] = 1 mg/L), while *qnrB52* and *qnrB91* caused high-level resistance (MIC = 8–16 mg/L). Notably, *qnrB91* reduced biofilm formation, indicating a trade-off between resistance and colonization. Growth assays showed that *qnrB52*, *qnrB91*, and *qnrS1* inhibited normal growth, whereas *qepA1* and *qnrS1* enhanced growth under ethanol stress. Most PMQR genes (except *qnrB6*) attenuated bacterial adhesion. *qepA1* promoted intracellular survival in macrophages, suggesting a role in chronic infection. Animal models confirmed that *qnrB6*, *qnrB7*, *qnrVC6*, and *aac(6*′*)-Ib-cr* significantly enhanced virulence. This study is the first in China to report *qnrVC6* and novel *gyrA* mutations (Ser83Ala/Val, Asp87Phe/His) in *K. pneumoniae*. It systematically reveals how PMQR genes influence infection by modulating resistance, immune evasion, and pathogenicity. These findings highlight that PMQR genes contribute not only to antibiotic resistance but also to virulence, suggesting that treatment strategies should consider specific PMQR genotypes. This research provides the largest-scale molecular epidemiological data and a theoretical basis for controlling quinolone-resistant *K. pneumoniae* in China.

**IMPORTANCE:**

Quinolone-resistant *Klebsiella pneumoniae* poses a serious threat to public health, yet the role of plasmid-mediated quinolone resistance (PMQR) genes beyond antibiotic resistance remains underexplored. In this largest-scale multicenter study in China, we analyzed 2,433 clinical isolates and discovered that PMQR genes do more than just confer drug resistance—they also influence bacterial growth, stress survival, biofilm formation, and the ability to evade or persist within host immune cells. Some PMQR genes even enhance virulence in an animal model. These findings challenge the traditional view of resistance genes as mere contributors to drug failure, revealing that they can also shape infection outcomes by altering bacterial behavior. Understanding these dual roles may guide more precise treatment strategies targeting specific PMQR genotypes.

## INTRODUCTION

*Klebsiella pneumoniae*, a member of the Enterobacteriaceae family, is a common opportunistic pathogen that frequently colonizes the nasopharynx and gastrointestinal tract of healthy individuals (1). It has emerged as a major clinical and public health threat worldwide. *K. pneumoniae* can acquire antibiotic resistance genes through various mechanisms, leading to the emergence of multidrug-resistant (MDR) and extensively drug-resistant (XDR) strains (2). According to the World Health Organization (WHO) (3), antibiotic-resistant hypervirulent *K. pneumoniae* (hvKP) is becoming increasingly prevalent. The convergence of drug resistance and high virulence in some strains poses significant challenges to public health.

Quinolones are an important class of synthetic antimicrobial agents, categorized into four generations based on their structural features and antibacterial spectra. Among them, second-generation quinolones, distinguished by a fluorine atom at the six-position of their molecular structure, exhibit significantly enhanced antibacterial activity (4). This modification not only broadens their spectrum but also improves their pharmacokinetic properties, contributing to their widespread global use. However, with increasing clinical application, resistance to quinolones has become a serious concern in pathogens such as *K. pneumoniae*.

Quinolones target two type II topoisomerases: DNA gyrase and topoisomerase IV. Both enzymes play essential roles by catalyzing the double-strand breakage and rejoining of bacterial chromosomal DNA (5). Quinolones form a stable ternary complex with the enzyme and DNA, inducing conformational changes that disrupt the breakage-reunion cycle, ultimately leading to permanent DNA breaks and bacterial cell death. DNA gyrase is a tetramer composed of two *gyrA* and two *gyrB* subunits, while topoisomerase IV consists of two ParC and two ParE subunits (6). Mutations in any of the genes encoding *gyrA*, *gyrB*, *parC*, or *parE* may confer quinolone resistance. A specific region within *gyrA* and *parC*, known as the quinolone resistance-determining region (QRDR), is particularly associated with resistance and is where most relevant mutations occur. Currently, mutations in DNA gyrase and topoisomerase IV are the primary mechanisms of quinolone resistance (7). The presence of two or more mutations often results in high-level resistance to quinolones (8).

Plasmid-mediated quinolone resistance (PMQR) mechanisms mainly involve qnr proteins, the aac(6′)-Ib-cr acetyltransferase, and efflux pump systems (mainly qepA and oqxAB pumps). Qnr proteins bind directly to DNA gyrase and topoisomerase IV, shielding the enzymes from quinolone inhibition and thereby reducing their bactericidal activity (9). The *aac(6*′*)-Ib* gene encodes an aminoglycoside acetyltransferase that inactivates aminoglycoside antibiotics. Its variant, *aac(6*′*)-Ib-cr*, can also acetylate the piperazinyl ring of certain quinolones, conferring dual resistance to both antibiotic classes (10). The efflux pumps *qepA* and *oqxAB* reduce intracellular concentrations of quinolones by actively pumping them out of the bacterial cell (11). Although PMQR genes typically confer low-level resistance (12), their ability to spread via plasmids among bacterial strains significantly facilitates the dissemination of quinolone resistance. The synergy among these resistance mechanisms exacerbates the clinical challenge of quinolone resistance, underscoring the urgent need for enhanced resistance surveillance and the development of novel antibacterial strategies.

## MATERIALS AND METHODS

### Bacterial strains and growth conditions

A total of 2,433 strains of *Klebsiella pneumoniae* were collected between 2018 and 2022 from 37 hospitals across multiple regions in China, including East, North, South, Southwest, and Northeast China. The isolates were derived from four types of infections: bloodstream infections, respiratory tract infections, urinary tract infections, and intra-abdominal infections. Only the first isolate from each patient was included to avoid duplication, resulting in a total of 2,433 non-redundant clinical isolates.

All isolates were cultured in 5 mL of Luria-Bertani (LB) broth at 37°C with shaking at 200 rpm. Whole-genome sequencing (WGS) was employed for genomic analysis to confirm the identity of the isolates as *K. pneumoniae*, with a focus on detecting plasmid-mediated quinolone resistance (PMQR) genes and chromosome-mediated quinolone resistance-associated mutations. All strains were preserved at −80°C using the cryopreservation disk method to maintain viability and genetic stability for subsequent research (13).

### Antimicrobial susceptibility testing

The antimicrobial susceptibility of *Klebsiella pneumoniae* clinical isolates was determined using the broth microdilution method (14). The minimum inhibitory concentration (MIC) was interpreted according to the Clinical and Laboratory Standards Institute (CLSI) guidelines (14). Isolates with a levofloxacin MIC ≥ 2 mg/L or a ciprofloxacin MIC ≥ 1 mg/L were classified as quinolone-resistant.

### Whole-genome sequencing

To elucidate the prevalence characteristics of PMQR genes and QRDR mutations in *Klebsiella pneumoniae*, whole-genome sequencing (WGS) was performed on all 2,433 collected isolates. The sequencing service was completed by Beijing Novogene Bioinformatics Technology Co., Ltd. Genomic DNA was first extracted from each isolate using the phenol-chloroform method (15), and its purity and concentration were then rigorously assessed using a NanoDrop 2000 spectrophotometer. The optimal values indicating high-quality DNA are 1.8 for *A*_260/280_ and 2.2 for *A*_260/230_, respectively. The DNA was then randomly fragmented for the construction of sequencing libraries, and whole-genome sequencing was conducted using the NovaSeq 6000 S4 PE150 XP platform (Illumina, San Diego, CA, USA) (16). Raw sequencing data were initially subjected to quality control and filtering with fastp, and *de novo* genome assembly was subsequently carried out using SPAdes.

### Construction of PMQR gene expression vectors

A high-copy plasmid, pUC19, was used to construct expression vectors carrying PMQR genes (including *qnrB1*, *qnrB2*, *qnrB4*, *qnrB6*, *qnrB7*, *qnrB52*, *qnrB91*, *qnrS1*, *qnrS2*, *qnrVC6*, *qepA1*, and *aac(6′)-Ib-cr*) derived from clinical *Klebsiella pneumoniae* isolates (17). These constructs were subsequently transformed into the *K. pneumoniae* strain QD110. The detailed procedures were as follows: (i) Gene Acquisition: Target PMQR genes were amplified by PCR using genomic DNA from clinical resistant strains as the template. The sequences of the primers used are listed in Table S1. (ii) Vector Construction: Purified PMQR gene fragments and the pUC19 plasmid were digested with restriction enzymes and ligated overnight at 16°C using T4 DNA ligase. (iii) Chemical Transformation: The ligation products were transformed into DH5α competent cells and plated on agar containing ampicillin (100 mg/L) to select positive clones. (iv) Recombinant Plasmid Extraction: Recombinant plasmids were extracted from positive clones using the EndoFree Plasmid Midi Kit (CW2105S, CWBIO). The extracted plasmids were then verified by Sanger sequencing. (v) Electrotransformation: Verified recombinant plasmids were introduced into QD110 via electroporation, and transformants were selected on LB agar plates supplemented with ampicillin. (vi) Validation: The successful integration of exogenous PMQR genes into the plasmid vector was confirmed by colony PCR (using M13-F/R primers) and Sanger sequencing. The minimum inhibitory concentration (MIC) of transformants was determined using the broth microdilution method (14) to validate their resistance phenotypes. An empty vector control group (QD110-pUC19) was included for subsequent experiments.

### Stress phenotype assay

Freshly cultured bacterial strains were adjusted to prepare a bacterial suspension with a concentration of 1 × 10^3^ cells. Subsequently, LB agar plates containing different concentrations of antimicrobial agents were prepared. The drugs and concentrations used in Fig. 2 were determined based on a previous report (18) and our preliminary experiments. Initial concentrations were obtained from the report, followed by preliminary experiments to select effective concentrations that partially, rather than completely, inhibited bacterial growth. A 10 µL aliquot of each bacterial suspension was spot-inoculated onto the drug-containing plates. Following incubation at 37°C for 12 h, bacterial growth was observed and photographed for documentation. The halos observed in the final experimental images are inherent to the photographic and imaging process. Similarly, the spotted background in the Deoxycholate-treated group is caused by the dissolution of this agent in the culture medium. These phenomena do not affect data interpretation or the reliability of the experimental conclusions.

### Bacterial growth determination

The biofilm formation assay was performed as described previously (19). A single colony was inoculated into LB liquid medium and cultured with shaking at 37°C until the logarithmic growth phase was reached (OD_600_ = 0.5). The bacterial suspension was then diluted 100-fold with fresh LB broth, and 200 μL of the diluted suspension was aliquoted into each well of a 96-well cell culture plate. The inoculated plate was incubated at 37°C for 24 h in a multifunctional microplate reader. The optical density at 600 nm was measured every 30 min using the microplate reader to dynamically monitor bacterial growth. All experiments were performed in triplicate, and growth curves were generated based on the collected data.

### Capsule quantification

Briefly (20), bacterial cultures were grown in LB medium until the OD_600_ = 1.0 ±  0.1. Then, 500 μL of the culture was mixed with 100 μL of a 100 mM citrate buffer (pH 2.0) containing 1% Zwittergent 3-14. The mixture was incubated at 50°C for 30 min with periodic mixing. After incubation, bacterial cells were pelleted by centrifugation at 16,000 × *g* for 5 min, and 250 μL of the supernatant was combined with 1 mL of anhydrous ethanol (final concentration 80%) and allowed to stand on ice for 30 min. The sample was centrifuged again at 16,000 × *g* for 5 min, and the supernatant was discarded. The pellet was air-dried at 37°C and resuspended in 100 μL of ddH_2_O. Next, 0.6 mL of a solution containing 12.5 mM sodium tetraborate in concentrated sulfuric acid was added, and the mixture was boiled at 100°C for 5 min, then cooled to room temperature. Subsequently, 20 μL of 0.15% 3-hydroxydiphenol solution (prepared in 0.5% sodium hydroxide) was added, and the absorbance was measured at 520 nm. A standard curve was generated using a concentration gradient of glucuronolactone standards, and the capsular polysaccharide content in the samples was quantified based on this standard curve.

### Siderophore detection

Quantification of siderophore production was performed using the Chrome Azurol S (CAS) assay (21). Briefly, 1 mL of an overnight LB culture grown at 37°C was centrifuged (5 min, 9,400 × *g*), and the pellet was collected. The pellet was washed twice with M9 minimal medium and resuspended in 1 mL of the same medium. Then, 50 μL of the resuspended bacteria were inoculated into 5 mL of fresh M9 minimal medium and incubated at 37°C until the OD_600_ reached approximately 1.3. The culture was placed on ice for 15 min, followed by centrifugation at 4,300 × *g* for 20 min at 4°C. The supernatant was collected and filtered through a 0.22 μm membrane. Next, 150 μL of the supernatant was mixed with 150 μL of CAS assay working solution and incubated at room temperature for 2 h in the dark. The absorbance at 630 nm was measured and recorded as *A*_1_. A negative control was prepared by mixing 150 μL of fresh M9 minimal medium with 150 μL of CAS working solution, and its absorbance was measured under the same conditions and recorded as *A*_0_. The siderophore production rate was calculated using the following formula: Siderophore Production (%) = [(*A_0_ − A*_1_)/*A*_0_] × 100%.

### Biofilm formation assay

The bacterial suspension, cultured to the logarithmic growth phase (OD_600_ = 0.5), was inoculated into a 96-well cell culture plate at 200 μL per well and incubated statically at 37°C for 24 h. After incubation, the supernatant was carefully aspirated, and the wells were gently washed three times with physiological saline to remove non-adherent planktonic bacteria. Subsequently, 0.1% crystal violet solution (wt/vol) was added and allowed to stain at room temperature for 15 min to label the biofilm structures. Following staining, the wells were thoroughly rinsed with physiological saline to remove unbound dye. Finally, 200 μL of absolute ethanol was added to dissolve the crystal violet bound to the biofilm, and the optical density at 600 nm was measured using a microplate reader to quantitatively assess biofilm formation capacity (22).

### Bacterial adhesion assay

Prior to the infection assay, A549 human carcinoma epithelial cells in the logarithmic growth phase were seeded into 12-well cell culture plates at a density of 5 × 10⁵ cells per well. Each well was supplemented with 1 mL of RPMI 1640 medium containing 10% fetal bovine serum (Gibco, Grand Island, New York, NY, USA), and the plates were incubated at 37°C under 5% CO_2_ for 16–24 h. Infection was carried out at a multiplicity of infection (MOI) of 50:1. After 2 h of infection, the cells were gently washed three times with phosphate-buffered saline (PBS) to thoroughly remove non-adherent bacteria. Subsequently, 1 mL of 0.1% Triton X-100 solution was added to each well to lyse the cells for 30 min. The lysates were collected, serially diluted, and plated onto LB agar plates for viable bacterial counting. The results were expressed as colony-forming units (CFU) per well (23).

### Macrophage phagocytosis assay

Prior to the infection assay, RAW 264.7 macrophage cells in the logarithmic growth phase were seeded into 12-well tissue culture plates at a density of 5 × 10⁵ cells per well. Each well was supplemented with 1 mL of Dulbecco’s modified Eagle medium (DMEM) containing 10% fetal bovine serum (FBS), followed by incubation at 37°C under 5% CO_2_ for 16–24 h. Infection was performed at a multiplicity of infection (MOI) of 100:1. After 2 h of infection, the medium was replaced with DMEM containing 100 mg/L colistin and incubated for an additional 0.5 h to thoroughly eliminate extracellular bacteria. The cells were then washed three times with phosphate-buffered saline (PBS), lysed with 1 mL of 0.1% Triton X-100 solution per well for 30 mins, and the resulting lysates were serially diluted and plated onto LB agar plates for viable bacterial counting. The results were expressed as colony-forming units (CFU) per well (24).

### Intracellular bacterial survival assay

RAW 264.7 macrophages were seeded in cell culture plates at a density of 5 × 10⁵ cells per well and infected with bacteria at a multiplicity of infection (MOI) of 100, following the same procedure as described for the phagocytosis assay. This experiment included five time points (0, 1, 2, 4, and 6 h) with three replicate wells per time point. After 0.5 h of infection, the medium was replaced with DMEM containing 100 mg/L colistin E and incubated for an additional 0.5 h to thoroughly eliminate extracellular bacteria, followed by three washes with PBS. This time point was designated as “0 h” at which one set of samples was immediately collected by adding 1 mL of 0.1% Triton X-100 to lyse the cells for 30 min, and the intracellular bacterial count was determined as the baseline value. The remaining five sets of samples were replenished with 1 mL of fresh complete DMEM medium and further incubated until their respective time points (1, 2, 4, and 6 h). At each designated time point, the corresponding sample was collected, lysed with 0.1% Triton X-100. After the cells are lysed to release intracellular bacteria, the lysate is serially diluted, and 10 μL of each dilution is plated on LB agar plates. Plates with countable colonies are selected, and the total number of bacteria can be calculated by multiplying the number of colonies by the corresponding dilution factor.

### Galleria mellonella virulence assay

This study employed the *Galleria mellonella* larval infection model for *in vivo* virulence assessment (25). Healthy final-instar larvae were randomly assigned to experimental groups, with 10 larvae per group placed in sterile Petri dishes. Bacterial suspensions of the test strains were adjusted to a concentration of 1 × 10^6^ CFU/mL using phosphate-buffered saline (PBS). Larvae were gently immobilized to expose the abdominal surface, and 10 μL of the bacterial suspension (corresponding to an actual inoculum of 10^3^ CFU per larva) was slowly injected into the second left proleg using a microsyringe. Following inoculation, all larval groups were incubated at 37°C in the dark. Survival status was systematically recorded at 12-h intervals over a 72-h period post-infection. Larval death was determined by the criteria of complete melanization and absence of response to physical stimulation. Survival curves were generated based on the observational data, and the relative pathogenicity of each test strain was evaluated using the standard hypervirulent strain NTUH-K2044 as a positive control.

## RESULTS

### Source and distribution of isolates

From 2018 to 2022, a total of 2,433 *Klebsiella pneumoniae* isolates were obtained from clinical specimens collected across 37 hospitals in China. The clinical sources included bloodstream infections, respiratory tract infections, urinary tract infections, and intra-abdominal infections. Bloodstream infections were the most common source (*n* = 1,040, 42.7%), followed by respiratory tract infections (*n* = 481, 19.7%), urinary tract infections (*n* = 461, 18.9%), and intra-abdominal infections (*n* = 451, 18.5%).

### Prevalence of fluoroquinolone resistance and resistance genes in *Klebsiella pneumoniae*

The minimum inhibitory concentrations (MICs) of levofloxacin against 2,433 *Klebsiella pneumoniae* isolates were determined using the broth dilution method (14), with susceptibility interpretation following CLSI guidelines. According to antimicrobial susceptibility testing results, among the 2,433 *K. pneumoniae* isolates, the resistance rate (MIC ≥ 2 mg/L) to levofloxacin was 44.43% (*n* = 1,081), the intermediate rate (MIC = 1 mg/L) was 9.16% (*n* = 223), and the susceptibility rate (MIC ≤ 0.5 mg/L) was 46.40% (*n* = 1,129).

**Fig 1:**
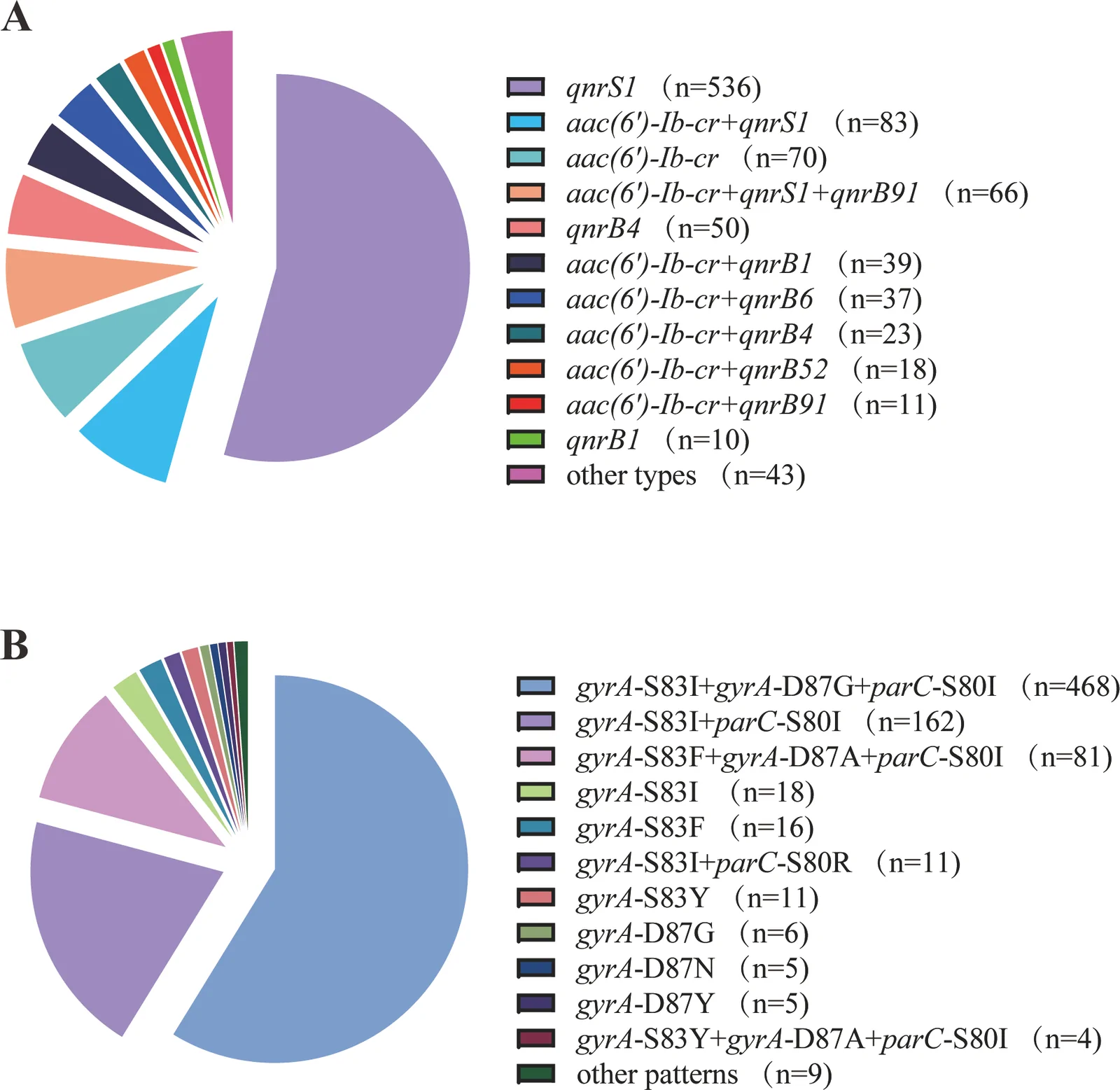
(**A**) Presence of combination types of PMQR genes in 986 *Klebsiella pneumoniae* strains carrying PMQR genes. The predominant combination types were *qnrS1* (*n* = 536), *aac(6*′*)-Ib-cr + qnrS1* (n = 83), *aac(6*′*)-Ib-cr* (*n* = 70), and *aac(6*′*)-Ib-cr + qnrS1 + qnrB91* (*n* = 66). (**B**) QRDR mutation patterns in 796 *Klebsiella pneumoniae* strains with QRDR mutations. The three most prevalent mutation patterns were *GyrA*-S83I + *GyrA*-D87G + *ParC*-S80I (*n* = 468), *GyrA*-S83I + *ParC*-S80I (*n* = 162), and *GyrA*-S83F + *GyrA*-D87A + *ParC*-S80I (*n* = 81). A, alanine; D, aspartic acid; F, phenylalanine; G, glycine; I, isoleucine; N, asparagine; R, arginine; S, serine; Y, tyrosine.

Of all 2,433 *K. pneumoniae* isolates, 986 strains (40.53%) carried PMQR genes. Among the 1,304 levofloxacin-non-susceptible *K. pneumoniae* isolates, 972 strains carried at least one PMQR gene, with *qnrS*, *qnrB*, and *aac(6*′*)-Ib-cr* being the predominant types. The distribution of PMQR genes varied depending on the levofloxacin MIC level. The vast majority of PMQR genes were detected in non-susceptible groups, while their detection rate was very low in susceptible isolates (Table 1). Among the combination types of PMQR genes, the solitary presence of the *qnrS1* gene exhibits the highest prevalence, while the combination of *aac(6′)-Ib-cr* with *qnr* genes is also relatively common (Fig. 1A). No *qnrA*, *qnrC*, *qnrD*, and *qnrE* genes were identified in this study.

**TABLE 1:** Distribution of PMQR genes in 2,433 *Klebsiella pneumoniae* isolates grouped based on levofloxacin MIC

PMQR genes	Number of isolates							
	MIC ≤ 0.5	MIC = 1	MIC = 2	MIC = 4	MIC = 8	MIC = 16	MIC ≥ 32	Total
	(*n* = 1,129）	(*n* = 223)	(*n* = 158)	(*n* = 55)	(*n* = 83)	(*n* = 564)	(*n* = 221)	
*qnrB1*	0	13	4	0	6	27	5	55
*qnrB2*	0	1	0	0	0	0	0	1
*qnrB4*	0	20	9	3	2	34	19	87
*qnrB6*	0	6	11	3	0	17	6	43
*qnrB7*	0	0	0	0	0	6	0	6
*qnrB52*	0	0	1	1	3	20	5	30
*qnrB91*	0	1	1	0	9	59	13	83
*qnrS1*	8	129	100	30	39	266	136	708
*qnrS2*	0	0	1	0	0	0	0	1
*qnrVC6*	0	0	1	0	0	0	0	1
*aac(6*′*)-Ib-cr*	5	42	49	9	37	178	55	375
*qepA1*	1	0	0	0	0	0	0	1

Additionally, among all *K. pneumoniae* isolates, 32.7% (*n* = 796) exhibited QRDR mutations (involving *GyrA* and *ParC*), of which 785 strains belonged to levofloxacin-non-susceptible *K. pneumoniae*. The primary mutation sites included: g*yrA*-Ser83 (→Ile, Phe, Tyr, Ala, Leu, Val), *gyrA*-Asp87 (→Gly, Ala, Asn, Tyr, Phe, His), *parC*-Ser80 (→Ile, Arg), and *parC*-Glu84 (→Lys). The most prevalent mutation pattern was *gyrA*-Ser83Ile + *gyrA*-Asp87Gly + *parC*-Ser80Ile (*n* = 468), followed by *gyrA*-Ser83Ile + *parC*-Ser80Ile (*n* = 162) and *gyrA*-Ser83Phe + *gyrA*-Asp87Ala + *parC*-Ser80Ile (*n* = 81) (Fig. 1B).

Among the 1,081 levofloxacin-resistant strains, 451 were isolated from bloodstream infections, 261 from urinary tract infections, 192 from abdominal infections, and 177 from respiratory infections. Regardless of the source, the predominant resistance type across all strains was the coexistence of PMQR genes and QRDR mutations, accounting for 41.67–55.86% of cases. Strains harboring only a single resistance factor (either PMQR genes or QRDR mutations) represented a secondary type. In contrast, strains lacking both factors constituted the lowest proportion, ranging only from 2.30% to 4.65%. These results suggest that, in addition to the known resistance mechanisms mentioned above, there may be other potential factors contributing to levofloxacin resistance in the strains. Based on QRDR mutations, we divided levofloxacin-resistant strains into five groups. The most prevalent category consisted of strains with double mutations in *GyrA* accompanied by a single mutation in *ParC* (*n* = 556), followed by strains in which no QRDR mutations were detected (*n* = 302), and strains harboring a single mutation in both *GyrA* and *ParC* (*n* = 173). Strains carrying only a single mutation in *GyrA* were less common (*n* = 49). Strains carrying double mutations in both GyrA and ParC were the rarest, with only one isolate identified, which tested negative for all PMQR genes. Additionally, among the levofloxacin-resistant strains, the more frequently observed PMQR gene combinations included *qnrS1*, *aac(6′)-Ib-cr + qnrS1*, *aac(6*′*)-Ib-cr + qnrS1 + qnrB91*, and *aac(6*′*)-Ib-cr* alone (Fig. S1).

Based on the aforementioned findings, our statistical analysis indicates a high prevalence of resistance to quinolone antimicrobial agents among clinical *Klebsiella pneumoniae* isolates in China. The widespread prevalence of PMQR genes, coupled with the accumulation of QRDR mutations, collectively drives the development and dissemination of levofloxacin resistance in *K. pneumoniae*. These findings provide critical epidemiological evidence for further elucidating the mechanisms underlying bacterial multidrug resistance.

### Comparison of the impact of PMQR genes on fluoroquinolone susceptibility in *Klebsiella pneumoniae*

Using the pUC19 plasmid vector, we introduced various PMQR genes into *Klebsiella pneumoniae* QD110 to systematically evaluate their effects on its biological functions. Compared to the empty vector control strain (QD110-pUC19), the presence of PMQR genes conferred a varying degree of increase in the MICs of fluoroquinolones for *Klebsiella pneumoniae* (Table 2). Strains carrying the *qnrB1*, *qnrB2*, *qnrB4*, *qnrB6*, *qnrB7*, *qnrS1*, or *qnrS2* genes exhibited an increase in MIC for levofloxacin and ciprofloxacin to 1 and 2 mg/L, respectively. Strain carrying the *qepA1* gene showed an increase in MIC for both drugs to 0.25 and 0.5 mg/L, respectively. In contrast, strain carrying the *aac(6*′*)-Ib-cr* gene only exhibited an increase in MIC for ciprofloxacin to 0.25 mg/L. Notably, *qnrB52* and *qnrB91* demonstrated the most significant enhancement in resistance. The strain carrying *qnrB52* exhibited MICs of 8 mg/L against both fluoroquinolones, while the strain carrying *qnrB91* showed even higher MICs of 16 mg/L. Sequence analysis revealed that both *qnrB52* and *qnrB91* harbored a single nucleotide mutation at position 259, resulting in amino acid substitutions of Arg87Ser and Arg87Cys, respectively. This molecular alteration is likely the key mechanism underlying the marked increase in resistance.

**TABLE 2:** Fluoroquinolone susceptibility of *Klebsiella pneumoniae* carrying different PMQR genes

Strains	MIC (µg/mL)	
	LVX	CIP
QD110-*qnrS1*	1	4
QD110-*qnrS2*	1	2
QD110-*qnrB1*	1	2
QD110-*qnrB2*	1	2
QD110-*qnrB4*	1	2
QD110-*qnrB6*	1	2
QD110-*qnrB7*	1	2
QD110-*qnrB52*	8	8
QD110-*qnrB91*	16	16
QD110-*qnrVC6*	1	2
QD110-*aac(6*′*)-Ib-cr*	0.125	0.25
QD110-*qepA1*	0.25	0.5
QD110-pUC19	0.125	0.0625

### Impact of PMQR on the growth of *Klebsiella pneumoniae* under routine and stress conditions

**Fig 2:**
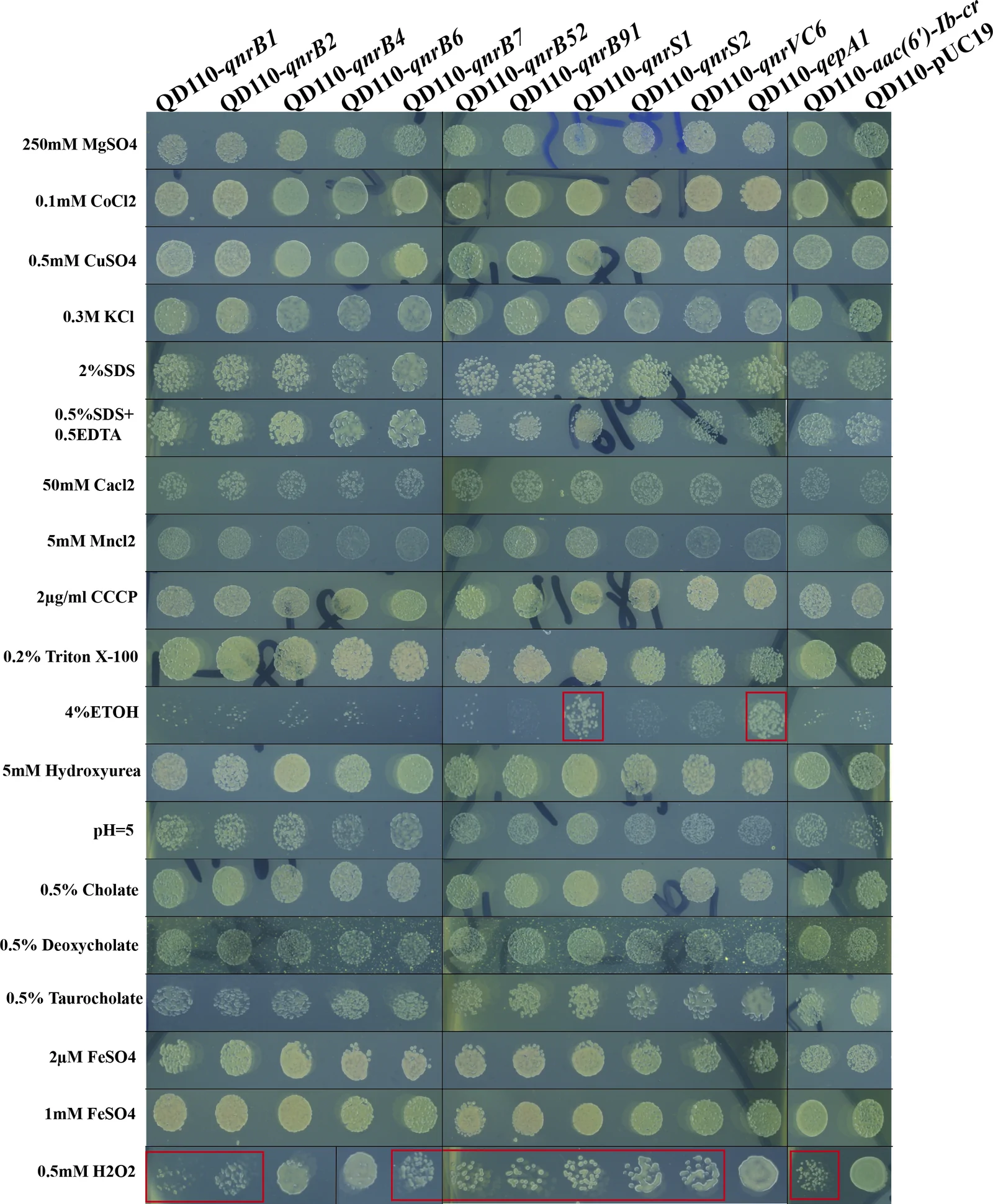
Growth of *Klebsiella pneumoniae* carrying different PMQR genes on agar plates under various antimicrobial pressure conditions. Of the 19 stress conditions, only ethanol and H_2_O_2_ exhibited a significant effect on the growth of the QD110 strain carrying different PMQR genes. CCCP, Carbonyl cyanide 3-chlorophenylhydrazone. The halos and background spots are natural phenomena and do not affect data interpretation.

**Fig 3:**
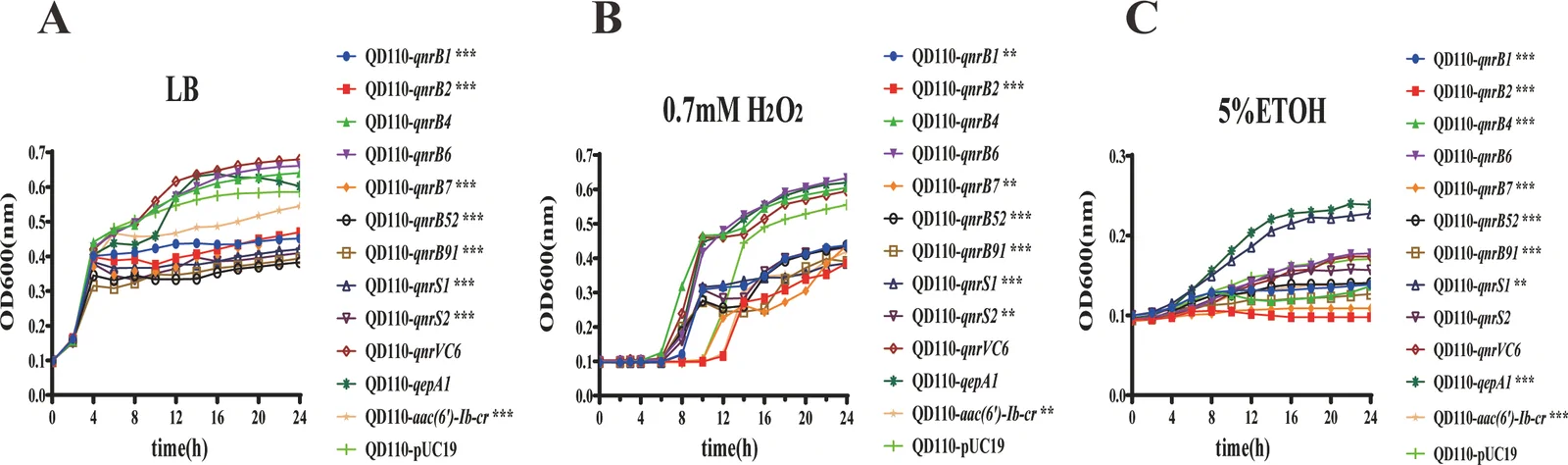
(**A–C**) Growth curves of Klebsiella pneumoniae QD110 carrying distinct PMQR genes in fresh LB broth, LB broth supplemented with 0.7 mM H_2_O_2_ or 5% ETOH. In LB broth and oxidative stress (0.7 mM H_2_O_2_) media, *qnrB4*, *qnrB6*, *qnrVC6*, and *qepA1* did not affect growth, while all other genes attenuated it. Under ethanol stress (5%), however, the effects diverged: *qnrS1* and *qepA1* promoted growth, *qnrB6*, *qnrS2*, and *qnrVC6* were neutral, and the rest were inhibitory. (*, *P* < 0.05; **, *P* < 0.01; ***, *P* < 0.001; ns, *P* > 0.05. Each experiment was performed three times and error bars represent standard deviation.)

Through experimental analysis of colony growth under a range of stress conditions, we identified two agents—ethanol and hydrogen peroxide (H_2_O_2_)—that significantly influenced bacterial growth (Fig. 2). Subsequent growth curve assays in standard LB broth showed that recombinant strains carrying *qnrB4*, *qnrB6*, *qnrVC6*, or *qepA1* exhibited growth comparable to that of QD110-pUC19 (Fig. 3A). In contrast, most other PMQR genes exerted varying degrees of growth inhibition.

Similarly, in an oxidative stress environment (0.7 mM H_2_O_2_), *qnrB4*, *qnrB6*, *qnrVC6*, and *qepA1* did not impair the growth fitness of *Klebsiella pneumoniae*. All other PMQR genes, however, resulted in significantly restricted growth (Fig. 3B).

Under ethanol stress (5% ethanol), strains QD110-*qepA1* and QD110-*qnrS1* demonstrated significantly enhanced growth rates compared to QD110-pUC19 (Fig. 3C). Meanwhile, *qnrB6*, *qnrS2*, and *qnrVC6* had no notable effect on growth, whereas the remaining PMQR genes markedly suppressed bacterial proliferation under this condition.

### Regulatory effects of PMQR on biofilm, capsule, and siderophore production in *Klebsiella pneumoniae*

**Fig 4:**
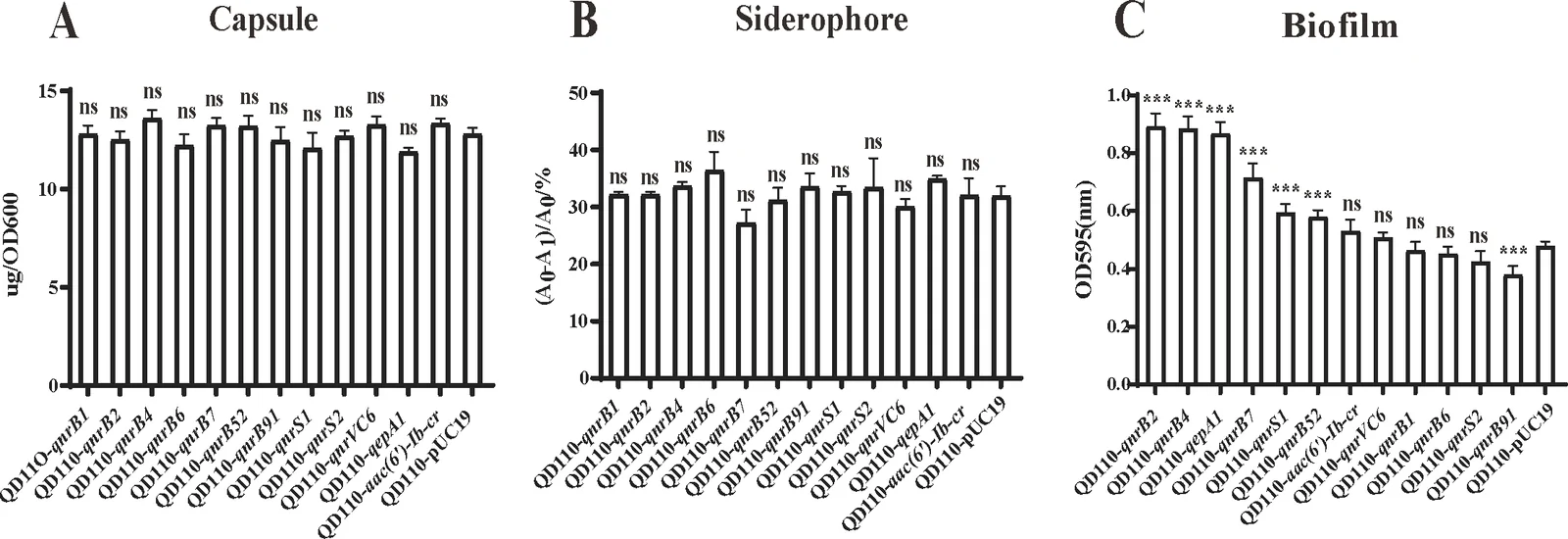
(**A**) There was no significant difference in capsule content among *Klebsiella pneumoniae* strains carrying different PMQR genes. (**B**) Similarly, no significant difference was observed in siderophore production across *Klebsiella pneumoniae* strains harboring various PMQR genes. (**C**) Biofilm formation capacity of *Klebsiella pneumoniae* QD110 harboring different PMQR genes. Several genes enhanced the biofilm formation capacity of QD110, with the effects of *qnr2*, *qnr4*, and *qepA1* being the most pronounced. (*, *P* < 0.05; **, *P* < 0.01; ***, *P* < 0.001; ns, *P* > 0.05. Each experiment was performed three times and error bars represent standard deviation.)

The impact of various PMQR genes on biofilm formation was quantitatively assessed using the crystal violet staining method. The results indicated that *qnrS1*, *qnrB2*, *qnrB4*, *qnrB7*, *qnrB52*, and *qepA1* significantly enhanced the biofilm synthesis capacity of the QD110 strain. In contrast, *qnrB91* markedly inhibited biofilm formation, while no other PMQR genes showed statistically significant effects (Fig. 4C). Regarding capsular polysaccharide content and siderophore production, the experimental results demonstrated that PMQR genes had no significant influence on either trait (Fig. 4A and B).

These findings suggest that PMQR genes are not only closely associated with quinolone resistance but may also broadly regulate core physiological functions of *Klebsiella pneumoniae*, including growth rate, stress response, and biofilm formation. Thereby, they influence the adaptability of *K. pneumoniae* during host infection.

### Effects of PMQR genes on the interaction between *Klebsiella pneumonia* and host cells

**Fig 5:**
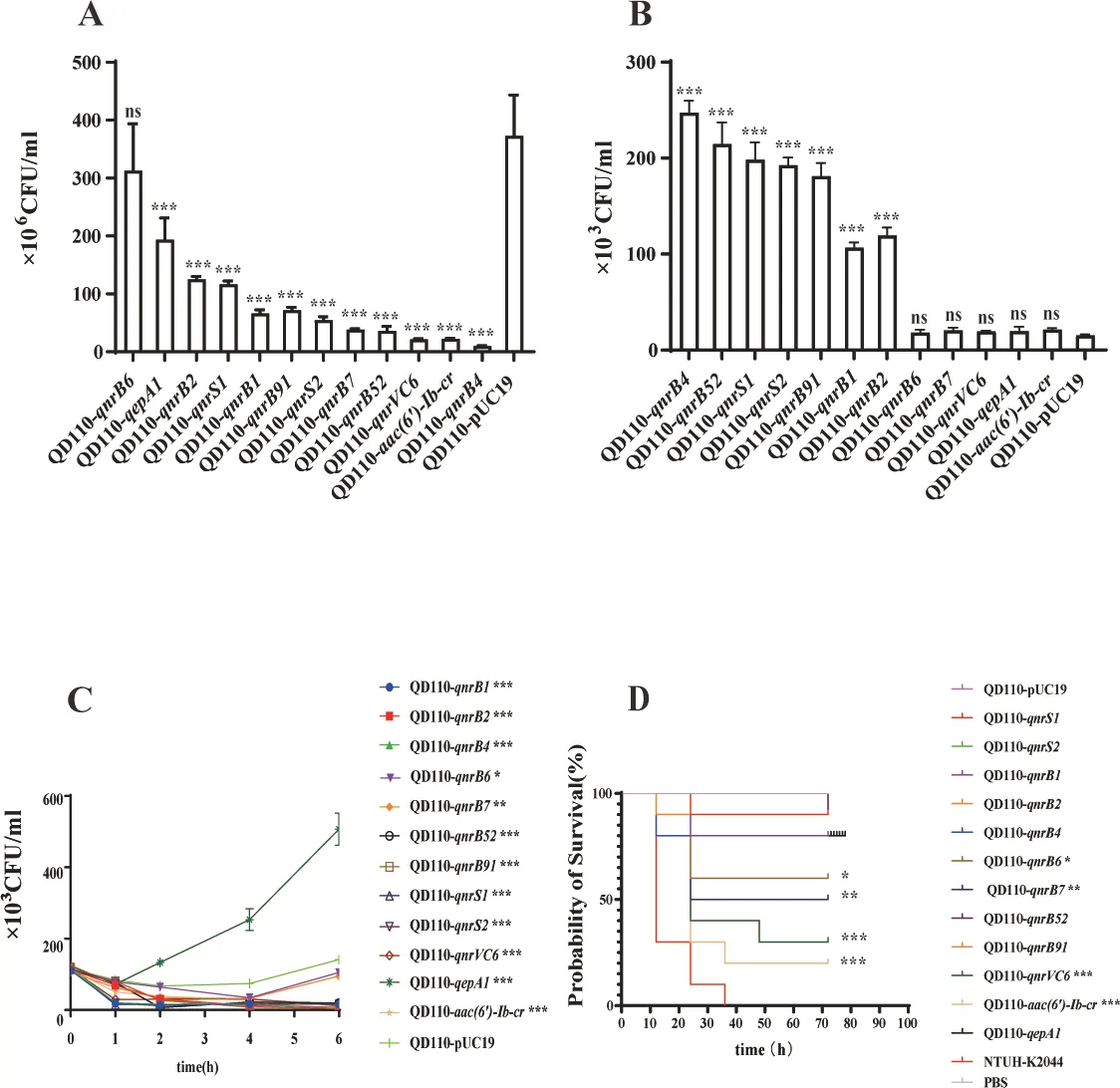
(**A**) Bacterial adhesion ability of QD110 strains carrying PMQR genes to A549 cells. Unlike *qnrB6*, which did not affect bacterial adhesion, all other tested genes significantly reduced the adhesion index of *Klebsiella pneumoniae*. (**B**) Effect of PMQR genes on the antiphagocytic ability of *Klebsiella pneumoniae* against RAW 264.7 macrophages. The genes *qnrB4*, *qnrB52*, *qnrS1*, *qnrS2*, *qnrB91*, and *qnrB1* significantly impaired the anti-phagocytic capability of the bacteria. (**C**) Intracellular survival ability of *Klebsiella pneumoniae* carrying PMQR genes in RAW 264.7 macrophages. With the exception of *qepA1*, all other PMQR genes were associated with a reduced intracellular survival ability in RAW 264.7 macrophages. (**D**) Compared to QD110-pUC19, strains carrying the *qnrB6*, *qnrB7*, *qnrVC6*, and *aac(6′)-Ib-cr* genes exhibited significantly enhanced pathogenicity toward *Galleria mellonella* larvae. All experiments used QD110-pUC19 as the control group for comparison. (*, *P* < 0.05; **, *P* < 0.01; ***, *P* < 0.001; ns, *P* > 0.05. Each experiment was performed three times and error bars represent standard deviation.)

This study investigated the effects of different PMQR genes on the interaction between *Klebsiella pneumoniae* QD110 and host cells through *in vitro* cellular experiments. First, we evaluated the impact of various PMQR genes on the bacterial adhesion ability to A549 cells. Bacterial adhesion assays revealed that (Fig. 5A), with the exception of *qnrB6*, all other tested PMQR genes significantly reduced the adherence of *K. pneumoniae* to A549 cells, indicating that most PMQR genes may negatively regulate initial colonization on epithelial cells.

To further explore the role of PMQR genes in immune evasion, we used a mouse macrophage cell line RAW264.7 model to assess the effects of different genotypes on bacterial anti-phagocytic ability and intracellular survival. Anti-phagocytosis assays demonstrated that (Fig. 5B), compared to the empty vector control strain, strains carrying *qnrB1*, *qnrB2*, *qnrB4*, *qnrB52*, *qnrB91*, *qnrS1*, and *qnrS2* exhibited significantly reduced anti-phagocytic capabilities, while no significant effects were observed for the other PMQR genes.

In the intracellular survival assay, a clear gene-dependent effect was observed (Fig. 5C). After 6 h of infection, the survival rate of strains carrying *qepA1* within macrophages was approximately four times higher than that of the control group, suggesting that this gene substantially enhances bacterial survival within host immune cells. In contrast, the intracellular survival rates of strains carrying *qnrB1*, *qnrB2*, *qnrB4*, *qnrB6*, *qnrB52*, *qnrB91*, *qnrS1*, *qnrS2*, *qnrVC6*, and *aac(6′)-Ib-cr* were significantly lower than those of the control, indicating that these genes may impair bacterial adaptability to survive within macrophages.

### Impact of PMQR on the virulence of *Klebsiella pneumoniae*

Compared to QD110-pUC19, larvae infected with strains carrying *qnrB6*, *qnrB7*, *qnrVC6*, or *aac(6*′*)-Ib-cr* exhibited significantly reduced survival rates, indicating that these genes considerably enhanced the pathogenicity of *K. pneumoniae*. Among these, QD110-*aac(6′)-Ib-cr* induced the highest larval mortality, demonstrating a particularly notable virulence-enhancing effect (Fig. 5D).

Taken together, our findings demonstrate that PMQR genes exert pleiotropic effects on *K. pneumoniae*, influencing not only antibiotic resistance but also key stages of host-pathogen interaction, including adhesion, phagocytosis evasion, and intracellular persistence. This study provides new experimental evidence for the multifaceted role of resistance genes in modulating bacterial pathogenicity, suggesting that PMQR genes may indirectly shape infection progression and outcomes by altering host-pathogen dynamics (Table S2).

## DISCUSSION

*Klebsiella pneumoniae* is a major global clinical and public health threat, and the WHO has assessed its risk of global transmission and infection as moderate. With the widespread clinical use of quinolone antibiotics, resistance to this drug class in *K. pneumoniae* has been progressively increasing. Our study conducted a systematic analysis of 2,433 *K. pneumoniae* isolates, revealing the epidemiological characteristics and resistance mechanisms of fluoroquinolone-resistant *K. pneumoniae*, as well as the impact of PMQR genes on bacterial fitness and pathogenicity, thereby providing important scientific evidence for clinical anti-infective therapy and the control of drug-resistant bacteria.

Antimicrobial susceptibility testing showed an overall resistance rate of 44.43% to levofloxacin, higher than data from CHINET—suggesting increasing clinical challenges posed by fluoroquinolone-resistant *K. pneumoniae*. At the molecular level, PMQR genes and QRDR mutations are the two primary mechanisms mediating levofloxacin resistance. Among the 1,304 levofloxacin-non-susceptible strains, 74.54% carried at least one PMQR gene. A total of 12 PMQR genes were detected, including *qnrB1*, *qnrB2*, *qnrB4*, *qnrB6*, *qnrB7*, *qnrB52*, *qnrB91*, *qnrS1*, *qnrS2*, *qnrVC6*, *qepA1*, and *aac(6*′*)-Ib-cr*, while *qnrA*, *qnrC*, *qnrD*, and *qnrE* were not detected. Among these, *qnrS, qnrB,* and *aac(6*′*)-Ib-cr* were the predominant genotypes, consistent with previous reports in other parts of Asia (26, 27), Iran (28), or the Arab countries (29). On the other hand, QRDR mutations occurred at a frequency of 32.7%, primarily at *gyrA*-Ser83 and *parC*-Ser80, with the triple mutation combination (*gyrA*-Ser83Ile + Asp87Gly + *parC*-Ser80Ile) being the most prevalent. To our knowledge, this study is the first to detect the *qnrVC6* gene in *K. pneumoniae* and to report the following amino acid substitutions in *gyrA*: Ser83Ala, Ser83Val, Asp87Phe, and Asp87His, although these mutations have been previously found in other species

Through *in vitro* functional experiments, this study further revealed the pleiotropic biological functions of PMQR genes in *K. pneumoniae*. In terms of drug susceptibility, different PMQR genes varied significantly in their ability to increase the MICs of fluoroquinolones. While PMQR genes typically confer low-level resistance, *qnrB52* and *qnrB91* (mutants of *qnrB2*) led to high-level resistance to levofloxacin and ciprofloxacin. The key substitution at Arg87 may promote resistance by altering protein conformation, enhancing DNA binding, or improving stability, providing potential targets for future inhibitor design aimed at qnr protein structures.

In conventional LB medium, the expression of most PMQR genes exerted an inhibitory effect on the growth of *Klebsiella pneumoniae*, suggesting that they may impose a certain metabolic burden on the host bacterium. However, the *qnrB4*, *qnrB6*, *qnrVC6*, and *qepA1* genes were exceptions, as they showed no significant impact on bacterial growth, indicating that these genotypes possess better adaptability under standard conditions. It is noteworthy that the influence of PMQR genes on growth exhibited condition-dependent variations under different stress environments. Under H_2_O_2_ conditions, the aforementioned four genes similarly did not negatively affect bacterial growth fitness. Particularly notable was that under ethanol stress conditions, strains carrying *qepA1* and *qnrS1* demonstrated a significantly enhanced growth rate compared to the control group, suggesting that these two genes may specifically improve the bacterium’s resistance to membrane disruption or protein denaturation stress. These results indicate that the fitness cost associated with PMQR genes is not constant, and under specific stress conditions that may be encountered during host infection, some PMQR genes can even confer a growth advantage that promotes bacterial survival.

Regarding biofilm formation, *qnrS1*, *qnrB2*, *qnrB4*, *qnrB7*, *qnrB52*, and *qepA1* significantly enhanced the biofilm synthesis capacity of the strains, which may facilitate bacterial colonization and persistence on medical devices or host surfaces. In stark contrast, the high-level resistance gene *qnrB91* markedly inhibited biofilm formation, suggesting a potential "trade-off" in the regulation of collective behaviors by different PMQR genes.

This study provides in-depth insights into the complex roles of PMQR genes in host-pathogen interactions. One prominent finding is that the vast majority of PMQR genes (with the exception of *qnrB6*) significantly reduced bacterial adhesion to A549 epithelial cells. This appears to be disadvantageous for the initial establishment of infection. However, the effects of different genes diverged markedly in subsequent stages of immune evasion and intracellular survival. On one hand, several *qnrB* and *qnrS* gene variants resulted in decreased anti-phagocytic ability, making the bacteria more susceptible to clearance by macrophages. On the other hand, the *qepA1* gene exhibited a unique survival advantage; while it did not affect anti-phagocytosis, it increased its survival rate within macrophages by approximately fourfold, significantly enhancing its ability to persist within host immune cells. In contrast, most other PMQR genes impaired the bacterial capacity for intracellular survival.

Although *qnrB91* confers the highest level of fluoroquinolone resistance, its expression significantly inhibits biofilm formation, revealing an adaptive trade-off between the acquisition of high-level resistance and colonization capacity. In contrast, while *qepA1* only provides the host bacterium with a moderate resistance phenotype, it markedly enhances bacterial survival within macrophages (approximately fourfold increase in survival rate), suggesting that this gene may play a key role in promoting chronic and recurrent infections. It is noteworthy that in the *Galleria mellonella* infection model used in this study, the carriage of *qnrB6*, *qnrB7*, *qnrVC6*, and *aac(6′)-Ib-cr* significantly enhanced the virulence of *Klebsiella pneumoniae*, with *aac(6′)-Ib-cr* exhibiting the most pronounced virulence-enhancing effect. This finding reveals that these PMQR genes have the potential to increase pathogenicity in this specific infection model, suggesting a possible association with more severe clinical infection outcomes. However, due to the lack of direct clinical outcome data, this inference still requires further validation in future clinical studies. The expression level of the high-copy plasmid pUC19 used in our experiments differs from that of the low-copy plasmids typically found in clinical strains carrying PMQR genes. The primary objective of this study was to systematically compare the functional differences among various PMQR genes at a mechanistic level. Therefore, the experimental results should be regarded as a mechanistic validation of the potential biological effects of these genes, rather than being directly equated with an accurate representation of their common clinical manifestations in all clinical isolates.

This study systematically elucidates the multifaceted roles of PMQR genes in regulating antibiotic resistance and pathogenicity of *Klebsiella pneumoniae* from epidemiological and phenotypic perspectives. The results demonstrate that PMQR genes not only directly impact clinical outcomes by mediating antibiotic resistance, but more importantly, significantly influence bacterial environmental adaptability and pathogenic potential by regulating key biological processes such as bacterial growth, metabolism, stress response, biofilm formation, and host interactions. It should be noted that, although the high-copy system used in this study does not quantitatively recapitulate the typical plasmid copy number range in clinical isolates, it serves as a proof-of-principle model to evaluate the maximum potential fitness cost that could arise from PMQR gene overexpression. Given the difference in copy number between our system and native plasmids, the magnitude of this cost in clinical settings may be considerably lower than that observed here. Although their pathogenicity and virulence in humans require further validation through clinical studies, these findings highlight that the clinical impact assessment of PMQR genes should go beyond the limitations of traditional resistance profile analysis and require comprehensive consideration of their multi-layered regulation of overall bacterial fitness and pathogenic potential. Strains carrying specific PMQR genes may dominate the infection process through such comprehensive advantages. This understanding not only provides a new perspective for elucidating the prevalence and pathogenic mechanisms of drug-resistant strains, but also lays a theoretical foundation for developing novel anti-infection strategies targeting bacterial adaptive pathways.

## Data Availability

The sequence data reported in this paper have been deposited in the Genome Sequence Archive in National Genomics Data Center, China National Center for Bioinformation/Beijing Institute of Genomics, Chinese Academy of Sciences (GSA: CRA012126), that are publicly accessible at https://ngdc.cncb.ac.cn/gsa, and are also available from the corresponding author upon reasonable request.

## SUPPLEMENTAL MATERIAL

Figure S1 spectrum.01031-26-s0001.pdf Distribution of PMQR genes and QRDR mutations among 1,081 levofloxacin-resistant *Klebsiella pneumoniae* isolates.

Table S1 spectrum.01031-26-s0002.xlsx The primers used for the construction of recombinant plasmids.

Table S2 spectrum.01031-26-s0003.xlsx Summary table of all phenotypic assay results.

ASM does not own the copyrights to Supplemental Material that may be linked to, or accessed through, an article. The authors have granted ASM a non-exclusive, world-wide license to publish the Supplemental Material files. Please contact the corresponding author directly for reuse.
