# Supplementary material for: Molecular epidemiology of levofloxacin-resistant Klebsiella pneumoniae and the association of plasmid-mediated quinolone resistance genes with key biological phenotypes
Source: Microbiol Spectr. 2026 May 20;14(7):e01031-26. doi: 10.1128/spectrum.01031-26 (PMC13344114; doi:10.1128/spectrum.01031-26)
Supplement: Figure S1 — Distribution of PMQR genes and QRDR mutations among 1,081 levofloxacin-resistant Klebsiella pneumoniae isolates. [file spectrum.01031-26-s0001.pdf]

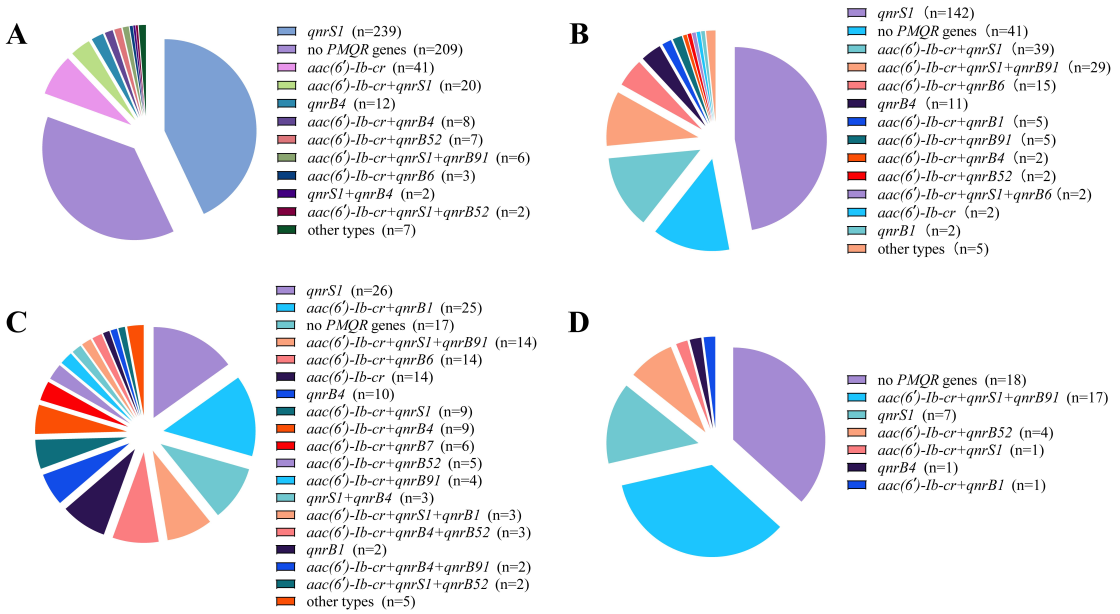

Supplementary Fig. S1 Distribution of PMQR genes and QRDR mutations among 1,081 levofloxacin-resistant *Klebsiella pneumoniae* isolates. **(A)** Distribution of PMQR genes among 556 strains with double mutations in *GyrA* accompanied by a single-site mutation in *ParC*; **(B)** Distribution of PMQR genes among 302 strains with no QRDR mutations; **(C)** Distribution of PMQR genes among 173 strains with a single mutation in both *GyrA* and *ParC*; **(D)** Distribution of PMQR genes among 49 strains with a single mutation in *GyrA*.
